# An Uncleaved Signal Peptide Directs the *Malus xiaojinensis* Iron Transporter Protein Mx IRT1 into the ER for the PM Secretory Pathway

**DOI:** 10.3390/ijms151120413

**Published:** 2014-11-07

**Authors:** Peng Zhang, Song Tan, James O. Berry, Peng Li, Na Ren, Shuang Li, Guang Yang, Wei-Bing Wang, Xiao-Ting Qi, Li-Ping Yin

**Affiliations:** 1 College of Life Science, Capital Normal University, Beijing 100048, China; E-Mails: zhangpeng_cnu@163.com (P.Z.); tansong_1515@163.com (S.T.); N.Naren@massey.ac.nz (N.R.); lishuanghebei@163.com (S.L.); ygemouse@163.com (G.Y.); wangweibing14@mails.ucas.ac.cn (W.-B.W.); qixiaoting@cnu.edu.cn (X.-T.Q.); 2 Department of Biological Sciences, State University of New York, Buffalo, NY 14260, USA; E-Mail: camjob@buffalo.edu; 3 School of Life Sciences, Tsinghua University, Beijing 100083, China; E-Mail: lipeng@tsinghua.edu.cn

**Keywords:** iron transport, *Malus xiaojinensis* iron-regulated transporter 1 (Mx IRT1), Mx DsIRT1, multi-pass membrane protein, metal uptake, uncleaved signal peptide, ER (Endoplasmic reticulum) targeting

## Abstract

*Malus xiaojinensis* iron-regulated transporter 1 (Mx IRT1) is a highly effective inducible iron transporter in the iron efficient plant *Malus xiaojinensis*. As a multi-pass integral plasma membrane (PM) protein, Mx IRT1 is predicted to consist of eight transmembrane domains, with a putative *N*-terminal signal peptide (SP) of 1–29 amino acids. To explore the role of the putative SP, constructs expressing Mx IRT1 (with an intact SP) and Mx DsIRT1 (with a deleted SP) were prepared for expression in *Arabidopsis* and in yeast. Mx IRT1 could rescue the iron-deficiency phenotype of an *Arabidopsis irt1* mutant, and complement the iron-limited growth defect of the yeast mutant DEY 1453 (*fet3fet4*). Furthermore, fluorescence analysis indicated that a chimeric Mx IRT1-eGFP (enhanced Green Fluorescent Protein) construct was translocated into the ER (Endoplasmic reticulum) for the PM sorting pathway. In contrast, the SP-deleted Mx DsIRT1 could not rescue either of the mutant phenotypes, nor direct transport of the GFP signal into the ER. Interestingly, immunoblot analysis indicated that the SP was not cleaved from the mature protein following transport into the ER. Taken together, data presented here provides strong evidence that an uncleaved SP determines ER-targeting of Mx IRT1 during the initial sorting stage, thereby enabling the subsequent transport and integration of this protein into the PM for its crucial role in iron uptake.

## 1. Introduction

Iron is an essential nutrient for plants and other organisms. It is critical for many processes in plants, including serving as a cofactor for redox enzymes involved in photosynthesis and respiration, as well as a catalyst for chlorophyll biosynthesis [1]. Although naturally very abundant in soils, iron is not easily absorbed by plant roots due to its insolubility under neutral or alkaline conditions [1]. Previous studies have used reverse genetics to demonstrate that iron-regulated transporter 1 (IRT1) is a major multi-pass plasma membrane (PM) importer responsible for iron uptake from soil under conditions of iron deficiency [2,3,4]. Most significantly, severe chlorosis and lethality due to reduced iron uptake have been observed in an *Arabidopsis irt1-1* knockout mutant [4]. The IRT1 protein family is widespread, occurring in a variety of dicots and monocots including but not limited to *Arabidopsis* [2], maize [5], rice [6], and *Malus xiaojinensis* [7]. A series of studies with *Arabidopsis* have provided significant new insights on the sorting, trafficking and localization of IRT1 within plant cells. Surprisingly, IRT1 has been found to be predominantly localized to the *trans*-Golgi network and early endosomes of root hairs [8]. Additional findings have demonstrated that members of the sorting nexin1 (SNX1) family of proteins are necessary for the correct trafficking of At (*Arabidopsis thaliana*) IRT1, and for modulating overall iron uptake activity [9]. A recently identified endosomal protein, FYVE1 (a phosphatidylinositol-3-phosphate-binding protein recruited to late endosomes), was found to control IRT1 recycling and polar delivery to the PM outer domain [10]. Together these findings indicate that efficient iron uptake mediated by IRT1 ultimately relies on precise intracellular trafficking of this essential transporter to the outer PM domain. However, specific protein sequences or domains within the IRT1 protein itself that interact with cellular trafficking components to meditate its transport to this domain are currently not well understood. It is known that two Lys residues located within a cytosol-exposed loop domain affect the ubiquitination, endocytosis, and degradation of IRT1 [8]. It is clear that further studies of the domain-based sorting signals are needed to understand how the complex sorting pathway of IRT1 functions, and how this pathway might be modified for improving iron uptake and bioavailability in plants.

Trafficking of multi-pass PM proteins such as IRT1 typically require an *N*-terminal signal peptide (SP) which enables their incorporation into the ER (endoplasmic reticulum) endomembrane transport system, and an internal membrane-anchor stop transfer sequence that delineates a transmembrane region. The ER-targeting SPs of such proteins vary somewhat in their primary sequence and length, but are typically 15–30 residues long, with polar or charged residues at the *N*-terminal, followed by a 6–15 amino acid region of hydrophobic residues and a polar *C*-terminal region containing the cleavage site for signal peptidase [11,12]. As these ER targeted proteins are being translated in the cytoplasm, the signal recognition particle (SRP) recognizes the newly synthesized *N*-terminal signal peptide, represses translation, and attaches the nascent protein-ribosome complex to an SRP receptor on the ER membrane. The SRP then disassociates, allowing translation to continue, and the protein is brought into the ER lumen through a translocator channel as it is being synthesized [11,13]. Typically, the SP will then be cleaved from the *N*-terminal protein by an ER-resident signal peptidase. This mature protein, lacking the signal sequence, is then trafficked through the ER and inserted into the PM. However, a very small minority of mature ER processed proteins located at the surface of animal cells have been shown to retain their intact *N*-terminal SP, such as CD18, the β-subunit of β_2_-integrins [14]. Multi-pass PM proteins that retain an uncleaved *N*-terminal signal peptides have not yet been reported in plants.

*Malus xiaojinensis* is an iron-efficient plant species in the genus Malus [15,16]. Its inducible iron transporter Mx *IRT1* has been shown to be highly expressed in roots under Fe deficiency, and is considered to be a key determinant of iron-efficiency for this species [7]. We report here that the mature ER-transported Mx IRT1 protein retains its *N*-terminal signal peptide when inserted into the PM. Analysis using the bioinformatics tool SignalP4.1 [17] indicates the presence of a putative SP with domains characteristic of a cleaved SP at *N*-terminal 1–29 amino acids of Mx IRT1. The goal of this study was to identify the function of this putative SP through complementation and location analysis, using transgene complementation of an *Arabidopsis IRT1* mutant and the iron uptake deficient yeast mutant *fet3fet4*. Findings presented here demonstrate that mature Mx IRT1 iron transporter retains the bona fide *N*-terminal SP that directs ER entry and transport through the PM secretory pathway to the cell surface. This study provides the first evidence for a functional uncleaved SP in plants.

## 2. Result and Discussion

### 2.1. Analysis of a Predicted Signal Peptide on the Malus xiaojinensis Iron-Regulated Transporter 1 (Mx IRT1) N-Terminus

**Figure 1:**
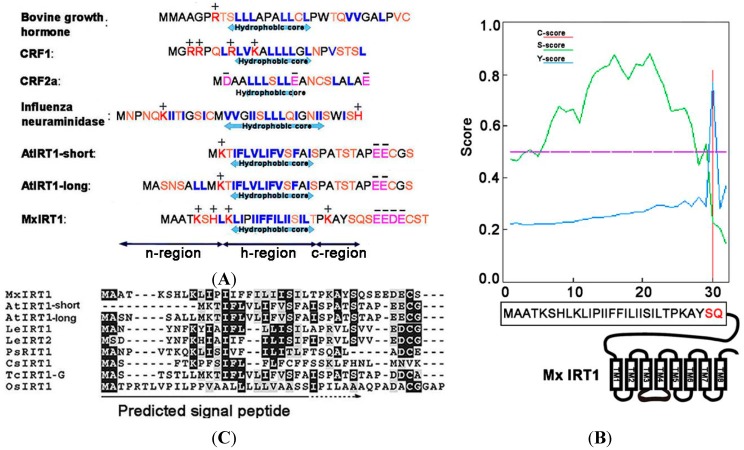
Prediction of the signal peptide (SP) on the *N*-terminus of *Malus xiaojinensis* iron-regulated transporter 1 (Mx IRT1). (**A**) Common features of ER (Endoplasmic reticulum)-processed SPs include a hydrophobic center region, surrounded by regions enriched in polar amino acids on both sides (blue: Hydrophobic amino acids; orange: Polar amino acids; red: Positive amino acids; and pink: Negative amino acids); (**B**) Mx IRT1 SP was predicted using SignalP4.1 [17]. *C*-score represents the raw cleavage site score (red); *S*-score indicates the SP score (green); and *Y*-score represents the combined cleavage site score (blue), which is a combination (geometric average) of the *C*-score and the slope of the *S*-score, providing a better cleavage site prediction than the raw *C*-score alone; and (**C**) Alignment of the predicted SP region with published IRT1 amino acid sequences, produced using ClustalW2 [19]. Identical residues are shown on a black background, and conservative substitutions are shown on a gray background.

The *N*-terminal SPs of ER-processed eukaryotic membrane and secretory proteins share similar canonical features, including a region containing polar and often charged amino acids at the *N*-terminus (*N*-region), a hydrophobic core (*H*-region) region at the center, and a polar region at the *C*-terminal (*C*-region) portion [11,18]. The SPs are typically cleaved from the mature protein during transport/processing through the ER. A comparative analysis of the *N*-termini of IRT1 proteins from several plant species with known cleavable SPs in other organisms indicated that these characteristic SP regions are present in the plant iron transport proteins as well (Figure 1A). For this study, we have focused primarily on the *N*-terminus of Mx IRT1. Using SignalP4.1 for analysis, it was found that amino acids located between the *N*-terminal and residue 30 of Mx IRT1 showed the highest *S*-scores, with a maximum of 0.882, and a mean *S*-score of 0.674. This indicates that the first 29 residues occur before the cleavage site. The Q residue at position 30 had a high *C*-score of 0.815 (Figure 1B), suggesting a likely cleavage site between the 29th (S:Ser) and 30th (Q:Gln) residues. These results are consistent with the presence of a cleavable 1–29 amino acid SP at the *N*-terminus of Mx IRT1 (Figure 1B).

While most *N*-terminal peptides with these characteristic features have been shown to be responsible for correct membrane sorting through the ER, and are cleaved from the mature protein during this process, there are some exceptions. For example, the *N*-terminus of the G protein-coupled receptor (GPCR) CRF2a (Figure 1A), predicted by SignalP [17] to possess features typical of a cleavable SP, has been found by functional analysis to a “*pseudo*” SP that cannot mediate ER trafficking and is not cleaved from the mature protein [20]. Furthermore, while SignalP4.1 has predicted that almost all IRT1 family members contain a putative cleavable *N*-terminal SP, an alignment of their actual amino acids sequences reveals only weak conservation (Figure 1C). Therefore, to understand the precise intracellular trafficking of Mx IRT1, it is important to confirm that its predicted *N*-terminal SP does in fact function as a bona fide, cleavable sorting peptide that mediates its ER trafficking, membrane localization, and ultimately its efficient iron uptake capability [8,10].

### 2.2. Deletion of the N-Terminal Mx IRT1 Signal Peptide (SP) Reduces Iron Uptake Ability in Arabidopsis and Yeast

**Figure 2:**
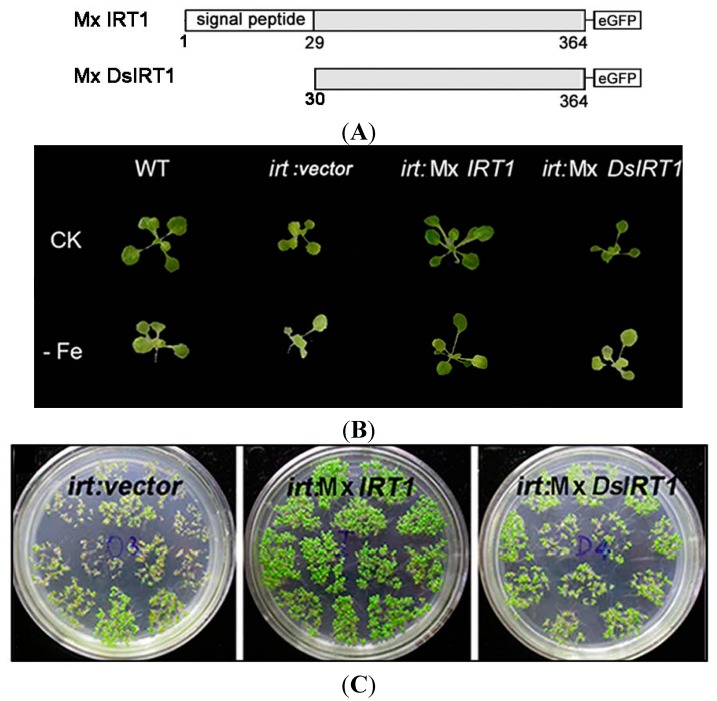

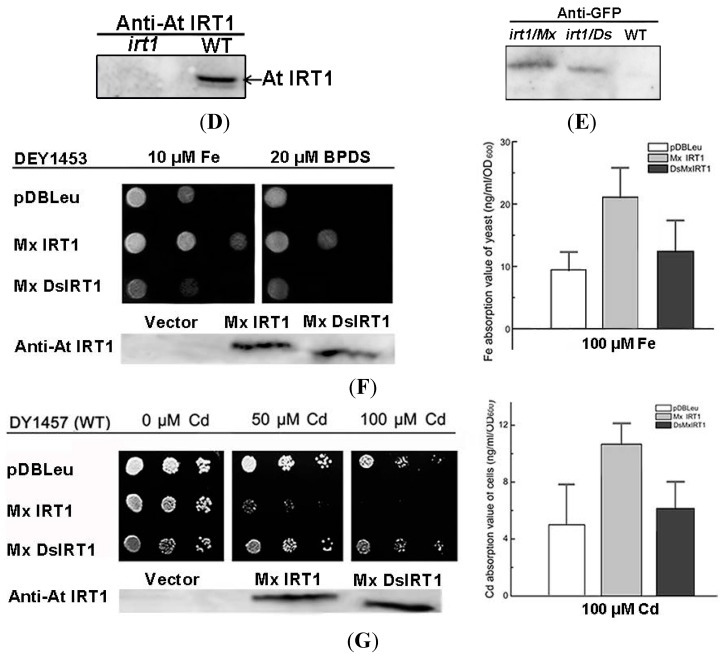
Transport ability of Mx IRT1 and Mx DsIRT1 in *Arabidopsis irt1* and yeast *fet3fet4* mutants. (**A**) Diagrams of the Mx IRT1-eGFP and Mx DsIRT1-eGFP constructs; (**B**) Phenotype analysis of WT (wild type) and *irt1* transformed lines; WT, *irt1*:*vector*, *irt1*:Mx *IRT1*, and *irt1*:Mx *DsIRT1* lines were grown under +Fe (CK) and −Fe conditions; (**C**) The germination and early growth comparisons for *irt1:vector*, *irt1:*Mx *IRT1*, and *irt1:*Mx *DsIRT1* lines. Seeds from each of the three lines were germinated and grown in 1/2 MS without iron (−Fe) for 5 days; (**D**) Immunoblot analysis showing the presence of At IRT1 in WT, but not in *Arabidopsis* *irt1* mutant plants. Proteins were harvested from plants after 3 days iron deficiency. The blots were probes with At IRT1 antibody; (**E**) Immunoblot analysis showing the presence of full length and truncated proteins in Mx IRT1-eGFP and Mx DsIRT1-eGFP transgenic lines, respectively, prepared by transforming *Arabidopsis IRT1* mutant plants. The blots were probed using GFP antibody; (**F**) Complementation and Immunoblotting analysis of Mx IRT1 and Mx DsIRT1 in DEY1453 (**left**); Blots probed with At IRT1 antisera show the presence of full length Mx IRT1 and truncated Mx DsIRT1 in the transformed yeast strains; the iron content was measured using inductively coupled plasma mass spectrometry (ICP-MS) after yeast cells had been cultured for 4 h in YPD (yeast extract peptone dextrose) liquid supplemented with 100 μM Fe^2+^-EDTA (ethylene diamine tetraacetic acid) (**right**); and (**G**) Cadmium sensitivity and Immunoblotting experiments in DY1457 (**left**); Blots probed with At IRT1 antisera show the presence of full length Mx IRT1 and truncated Mx DsIRT1 in the transformed yeast strains; and the cadmium content (**right**) of 3 transformed DY1457 was measured using ICP-MS after 100 μM treatment for 4 h.

To understand the role of the putative SP as a determinant of Mx IRT1 function, we used genetic complementation to comparatively analyze the metal uptake ability of *Arabidopsis* plants and yeast cells expressing a Mx IRT1, or a modified Mx IRT1 in which the predicted SP had been deleted (designated Mx DsIRT1) (Figure 2A). For expression in plants, these Mx *IRT1*, and Mx *DsIRT1* constructs were inserted into the expression vector pCAMBIA1301-35S_pro_-NOS_terminator_. A GFP (green fluorescent protein) tag at the *C*-terminal end of both proteins allowed for visualization of their trafficking through the plant cells by confocal fluorescence microscopy (please see Section 2.3). For complementation in yeast cells, these same constructs (without the GFP tag) were inserted into the expression vector pDBLeu. Genetic complementation/expression assays were performed using an *Arabidopisis irt1* iron uptake mutant, as well as the yeast mutant DEY 1453 (*fet3fet4*) that has impaired iron uptake ability.

Mx *IRT1* and Mx *DsIRT1* (and as a control, the vector alone) were introduced into an *Arabidopisis irt1* mutant that shows a lethal chlorotic phenotype associated with an iron deficiency condition. This severe chlorotic phenotype is not observed when WT (wild type) plants are grown under the same conditions. Furthermore, the *irt1* mutation can be complemented by constitutive expression of the endogenous At IRT1 from a 35S promoter (35S:At IRT1), with transformed plants showing the WT growth phenotype [3]. In this experiment, wild type (WT), *irt1*:vector (vector alone), *irt1*:Mx *IRT1*, and *irt1*:Mx *DsIRT1 Arabidopsis* lines were grown in 1/2 MS for 7 days, and then transferred to 1/2 MS medium supplemented with Fe (CK medium) or deficient in Fe (−Fe medium) for 10 days. On Fe-supplied media (CK), the growth of transgenic lines expressing the heterologous Mx IRT1 transporter was comparable to that of the WT seedlings (Figure 2B, top row). The growth of all four lines was affected to some degree on the Fe-media (Figure 2B, bottom row), and *irt1*:Mx *IRT1* transgenic lines (expressing the intact transporter) showed no significant differences from WT under these conditions. However, it is clear that the *irt1*:Mx *DsIRT1* transgenic lines (which lacked the *N*-terminal SP) displayed a strong chlorotic phenotype, with reduced greening and impaired leaf growth when compared to WT and *irt1*:Mx *IRT1* lines. The impaired growth phenotype of the SP-deficient Mx DsIRT1 plants was very similar to that of the control *irt1*:vector transgenic lines (which lacked the entire iron transporter) on the −Fe media (Figure 2B CK). In parallel, we examined seed germination and early growth (for five days) of the three transgenic lines on –Fe medium (Figure 2C). The *irt1*:Mx *IRT1* transgenic lines showed more efficient germination than the *irt1*:Mx *DsIRT1* and control *irt1*:vector lines, with substantially enhanced growth continuing over a period of five days. Western blot analyses confirmed that the endogenous At IRT1 protein accumulated in WT *Arabidopsis*, but not in untransformed *irt1* mutants. In the transgenic lines *irt1*: Mx *IRT1* and Mx *DsIRT1*, Western analysis confirmed accumulation of the Mx IRT1 and Mx DsIRT1, with little or no expression of the endogenous At IRT1 (Figure 2D,E). Collectively, these data demonstrate that expression of Mx ITR1 in transgenic *Arabidopisis irt1* mutants can complement the iron deficient phenotype. However, in lines expressing Mx DsIRT1, that lack the *N*-terminal SP, this phenotype is not complemented. Our finding that Mx IRT1 is not functional without its *N*-terminal SP establishes this region as an essential determinant for efficient iron transport in plants.

To further confirm that the *N*-terminal SP of Mx IRT1 is essential for iron uptake capability, we performed genetic complementation analysis in a heterologous system. Using the yeast expression plasmid pDBLeu, we introduced Mx IRT1 and Mx DsIRT1 constructs into the yeast mutant *fet3fet4* to determine if these proteins could rescue the iron-limited growth defect of this strain. In parallel, to definitively establish active iron uptake, we also measured the metal content of Mx *IRT1* and Mx *DsIRT1* transformed yeast mutant using inductively coupled plasma mass spectrometry (ICP-MS) [21].

As shown in Figure 2F, the Mx *IRT1* protein, containing an intact *N*-terminal SP, was able to rescue the *fet3fet4* mutant phenotype, enabling vigorous growth on media containing 10 mM Fe as well as on media containing the Fe chelator BPDS (bathophenanthroline disulfonic acid) (Fe deficient media). In comparison, the growth of yeast strains expressing the Mx *DsIRT1* (lacking the SP) and the empty vector was clearly impaired, most noticeably on the Fe-deficient media. Analysis of these cells using ICP-MS indicated that the strains transformed with Mx *DsIRT1* or empty vector accumulated much lower levels of iron than the strain transformed with the fully functional intact Mx *IRT1* when cultured in YNB liquid supplemented with 100 μM Fe^2+^-EDTA (ethylene diamine tetraacetic acid) (Figure 2F, right). Cadmium (Cd) sensitivity is another indicator of iron transport capability, since this metal is toxic to cells and brought in via iron transporters. Cd toxicity experiments showed that Mx *DsIRT1*-transformed lines grew much more vigorously than the Mx *IRT1*-transformed lines in the presence of higher cadmium concentration (Figure 2F, left, 50 and 100 µM Cd). Similar to Fe, the Cd content of Mx *DsIRT1* yeast strains was markedly lower than that of Mx *IRT1* strains after 100 μM Cd^2+^ treatment (Figure 2G, right). Western blot analyses of the *irt1*:Mx *IRT1* and Mx *DsIRT1* transformed yeast mutants, using At IRT1 antibody, confirmed the expression and accumulation of Mx IRT1 and Mx DsIRT1 protein within the transformed yeast lines (Figure 2F,G, left). In summary, and in agreement with findings from the genetic complementation of the *Arabidopsis* *irt1* mutant, findings presented here demonstrate that deletion of the *N*-terminal SP significantly reduced the metal uptake capability of Mx IRT1 in the heterologous yeast system, identifying this sequence as essential for the iron uptake capability of this transporter in both experimental systems.

### 2.3. Mx DsIRT1 Lacks Plasma Membrane (PM) Targeting Ability in Plant Cells and Yeasts

**Figure 3:**
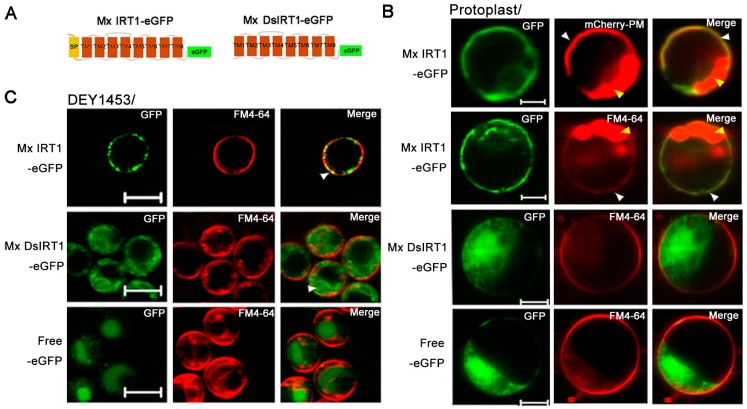

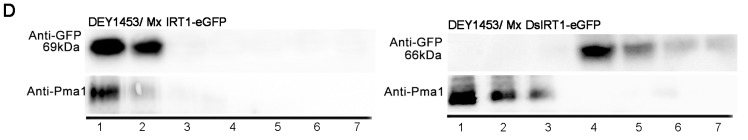
Localization and protein detection of Mx IRT1-eGFP and Mx DsIRT1-eGFP. (**A**) Diagrams of the Mx IRT1-eGFP and Mx DsIRT1-eGFP fusion proteins constructs used for localization analysis; (**B**) Images showing fluorescence localization of Mx IRT1-eGFP (**first and second rows**); Mx DsIRT1-eGFP (**third row**); and control free eGFP (**forth row**) in transformed protoplasts. The mCherry-PM (red) (**first row**) and FM4-64 (red) (**second row**) were pointed by the white arrow; the merge signals (yellow) (**first and second rows**) were also indicated by the white arrow; the strong red signal comes from the chloroplast autofluorescence, indicated by the yellow arrow; (**C**) Localization pattern of Mx IRT1-eGFP (**top row**); Mx DsIRT1-eGFP (**middle row**); and free eGFP (**bottom row**) in transformed yeast lines; Scale bar: 5 μm; and (**D**) Analysis of the different fractions (**1**–**7**) from the density gradient. Detection of Mx IRT1-eGFP (**left**) and Mx DsIRT1-eGFP (**right**) fusion proteins by Western blot using a GFP and a Pma1p antibody (Pma1p as the intact membrane protein marker).

Based on Mx *Ds*IRT1 reduced iron absorption capacity, we hypothesized that its *N*-terminus either plays a role in iron transport or guides Mx IRT1 to the PM for metal transport. To address the question, a GFP tag sequence was added to each of the transgenes, producing Mx IRT1-eGFP and Mx DsIRT1-eGFP fusion proteins. This tag allowed for a determination of their subcellular localization in plant protoplast and yeast cells by using fluorescent confocal microscopy (Figure 3A).

Both GFP-tagged fusion proteins were inserted into pBI221 under the control of the CaMV 35S promoter in pBI221. Mx IRT1-eGFP fusion proteins were transiently expressed in plant protoplasts. A partial co-localization (yellow) was observed between Mx IRT1-eGFP and mCherry-PM, the PM marker [22] by confocal microscopy 18 h after transfection (Figure 3B). Meanwhile, Mx IRT1-eGFP was distributed on the PM, as indicated by the presence of partial co-localization with FM4-64 (white arrow) (Figure 3B). In addition to the PM location of Mx IRT1-eGFP, the green signal was clearly observed intracellularly in the same protoplast (Figure 3B). However, Mx DsIRT1-eGFP and free eGFP were not observed at the PM, instead showing a wide dispersal pattern throughout the cytosol (Figure 3B, middle row). The dispersal pattern of the SP-deleted protein was very similar to the GFP alone control (Figure 3B, bottom row). These observations indicated that intact Mx IRT1 was targeted specifically to the PM, whereas Mx DsIRT1 was mis-sorted, showing the same non-specific cellular distribution pattern as the control.

**Figure 4:**
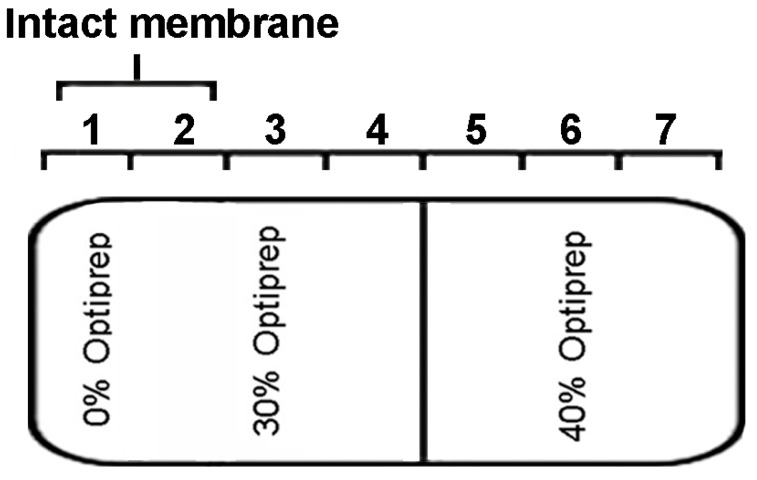
Optiprep density gradient centrifugation diagram. Intact membranes float at the interface between the 0% and 30% Optiprep, which corresponds to fraction **1** and **2**.

The vectors pYES-Mx *IRT1*-eGFP, pYES-Mx *DsIRT1*-eGFP, and the pYES-eGFP were transformed into the iron deficient yeast strain DEY1453 (*fet3fet4*). As shown in Figure 3C, Mx IRT1-eGFP also showed clear co-localization with FM4-64 in the PM (white arrow). In contrast, Mx DsIRT1-eGFP was spread throughout the cytosol in yeast. We then adopted the Optiprep density gradient centrifugation method to extract the intact membrane fraction from yeast in this study. The method was modified according to Bagnat *et al.* [23] and Mitsui *et al.* [24]. The proteins were isolated by density gradient centrifugation and the intact membrane floated in fraction 1 and 2 of the 0% to 30% Optiprep (Figure 4). Western blot analysis showed that Mx IRT1 was present in fractions 1 and 2, while Mx DsIRT1 protein was not detected (Figure 3E). Taken together, these results suggested that the lack of *N*-terminus disrupted the correct cellular localization of this iron transporter protein in both plant protoplast and yeast.

### 2.4. The N-Terminal 1–29 Amino-Acid Sequence Contributes to ER Targeting

Based on the effect of the Mx IRT1 SP deletion on its localization, and in view of previous findings that some PM membrane proteins are sorted to the PM through the SP-dependent ER secretory pathway [13,18,25], we systematically studied whether Mx IRT1 *N*-terminus is in fact an authentic SP capable of guiding the intact Mx IRT1 to the ER for synthesis and PM sorting.

**Figure 5:**
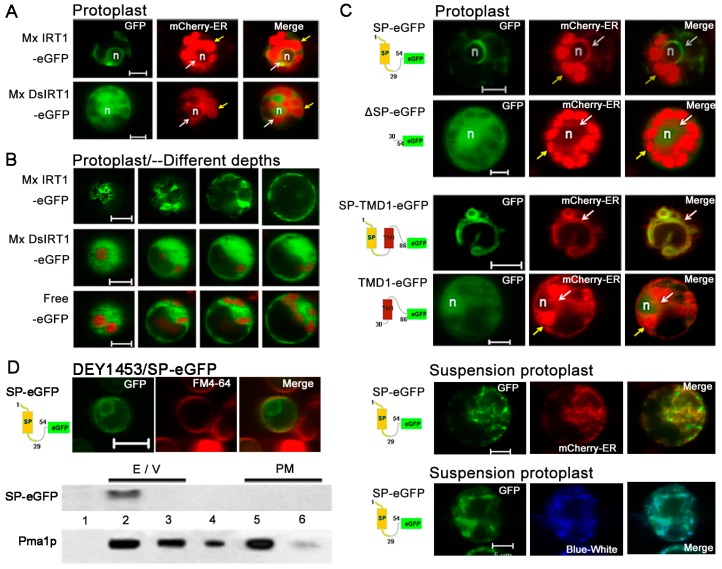
Comparative confocal fluorescent imaging shows localization of different GFP fusion proteins with or without an *N*-terminal SP in rice protoplast. (**A**) Images showing co-localization of Mx IRT1-eGFP (green) and mCherry-ER (red) (**top row**), with co-localization as yellow in the merged panel (white arrows); Mx DsIRT1-eGFP did not co-localized with the mCherry-ER (red) (**bottom row**). The strong red signal comes from the chloroplast autofluorescence, indicated by the yellow arrow; (**B**) Images at different depths of scanning in 3 transformed protoplasts; (**C**) Fluorescent localization of SP-eGFP (**1**–**54**), ΔSP-eGFP (**30**–**54**), SP-TM1-eGFP (**1**–**86**), and TM1-eGFP (**30**–**86**) in leafy protoplasts (marked by mCherry-ER) and suspension protoplast (marked by mCherry-ER (red) and ER- tracker Blue-White (blue)); and (**D**) Intracellular localization of SP (1–54)-eGFP in yeast; and western blot analysis of the ER/Vacuole (indicated by **2** and **3**) and PM fractions (indicated by **5** and **6**) from SP-eGFP transformed yeast using the GFP and Pma1p- antibodies. Scale bar: 5 μm. Note: The ER marker appears at a low intensity of red fluorescence, as indicated by the white arrow in **A** and **C**; the strong red signal comes from the chloroplast autofluorescence, as indicated by the yellow arrow. Scale bar: 5 μm; n: nucleus.

ER-mCherry (red) is a fluorescent marker protein that is targeted to the ER of plant cells [22]. When transiently expressed together with Mx IRT1-eGFP in protoplasts, both of these fluorescent labeled proteins were found to co-localize (yellow), showing a mesh-like pattern characteristic of ER localized proteins (Figure 5A, top row, white arrow indicates co-localization). This co-localization confirms that the full-length Mx IRT1 was guided to and translated in the ER. However, in the case of Mx DsIRT1-eGFP, the green eGFP fluorescence signal was detected diffusely throughout the cytoplasm, showing no co-localization with the mCherry-ER. The lack of co-localization indicates that the SP-deleted Mx DsIRT1-eGFP did not enter the ER for translation and sorting (Figure 5A, bottom row). For a more precise comparative determination of Mx IRT1-eGFP and Mx DsIRT1-eGFP distribution in protoplast, we used laser scanning for visualization of fluorescence at different cell depths using multiple focal planes (depth increases from left to right in Figure 5B). This multi-focal plane scanning revealed that a mesh-like structure associated with Mx IRT1-eGFP occurred throughout the cell (Figure 5B, top row), while the Mx DsIRT1-eGFP was evenly distributed throughout the cytosol in a highly diffuse pattern (Figure 5B, middle row), in a pattern indistinguishable from the eGFP alone (Figure 5B, bottom row). From the Figure 3B and Figure 5A,B, we found the Mx IRT1-eGFP localized both to the ER and the PM in the same cell. As reported, At IRT1 was also observed both in the intracellular compartment and PM in the same root hair cell [8]. Interestingly, At IRT1 accumulated under metal-depleted conditions at the outer polar domain of the PM facing the rhizosphere [10]. These results indicate the IRT1 protein experiences a dynamic location according to the environmental change, which process may control ion absorption. Therefore, it is conceivable that the significant accumulation of Mx IRT1in the ER may keep the rate of export from this compartment for the balance of iron uptake.

To confirm whether the putative SP is the bona fide ER-signal peptide, we designed the SP_(1–54)_-eGFP, and ΔSP-eGFP_(30–54)_ (negative control) constructs and incorporated these into plant expression vectors (Figure 5C) (see Table S2 for fusion protein sequences). A putative functional SP should direct the soluble eGFP marker to the ER. If the sequence does not contain a functional signal peptide, the eGFP should remain in the cytosol as free eGFP. As shown in Figure 5C, the SP_(1–54)_-eGFP signal (green) was clearly observed in the inner membrane system, and it co-localized with the mCherry-ER marker. These data showed that the *N*-terminal SP successfully targeted eGFP to the ER, but the ΔSP_(30–54)_-eGFP and Mx DsIRT1 were dispersed in the cytoplasm (Figure 5C). According to a previous report, the TM1 of CFR2a functions as an ER-signal sequence [20]. We subsequently constructed SP-TM1-eGFP_(1–86)_ and TM1-eGFP_(30–86)_ (Table S2) to investigate whether other transmembrane domains also function as the ER-signal sequence. The SP-TM1-eGFP_(1–86)_ signal remained in the ER, but TM1-eGFP_(30–86)_ was dispersed in the protoplast cytosol, indicating the TM1 was unable to mediate ER targeting (Figure 5C). In order to avoid the problem of chloroplast autofluorescence, we performed our experiments in cell suspension protoplast. The mCherry-ER and ER-Tracker™ Dyes Blue-White DPX were used to specifically label the ER. It showed that the SP_(1–54)_-eGFP clearly co-localized with the mCherry-ER and Blue-White DPX.

An additional SP_(1–54)_-eGFP expression construct was prepared for observation of SO-mediated protein trafficking in yeast cells. The SP_(1–54)_-eGFP presented the same localization as in protoplasts (Figure 5D). For further analysis, ER/vacuole and PM fractions were purified from pYES-SP_(1–54)_-eGFP transformed yeast by using sucrose density gradient centrifugation, and evaluated by immunoblot analysis (Figure 5D). SP_(1–54)_-eGFP was identified primarily within the ER/vacuole fractions, with two distinct bands observed. As a positive control, the membrane-associated Pma1p protein was found by immunoblot analysis to be localized to all of the membrane fractions, mainly in PM fraction (Figure 5D, bottom row).

Taken together, these data are support the hypothesis that the *N*-terminal 1–29 amino-acid sequence of Mx IRT1 functions as the bona fide ER-signal peptide that directs targeting of this iron transport to the ER membrane system, with its functionality confirmed in both plant and yeast cells. This data is in accordance with the canonical model of *N*-terminal SP sequences that are required for targeting PM proteins to the ER.

### 2.5. The SP Remains on the Mature Mx IRT1 Protein

**Figure 6:**
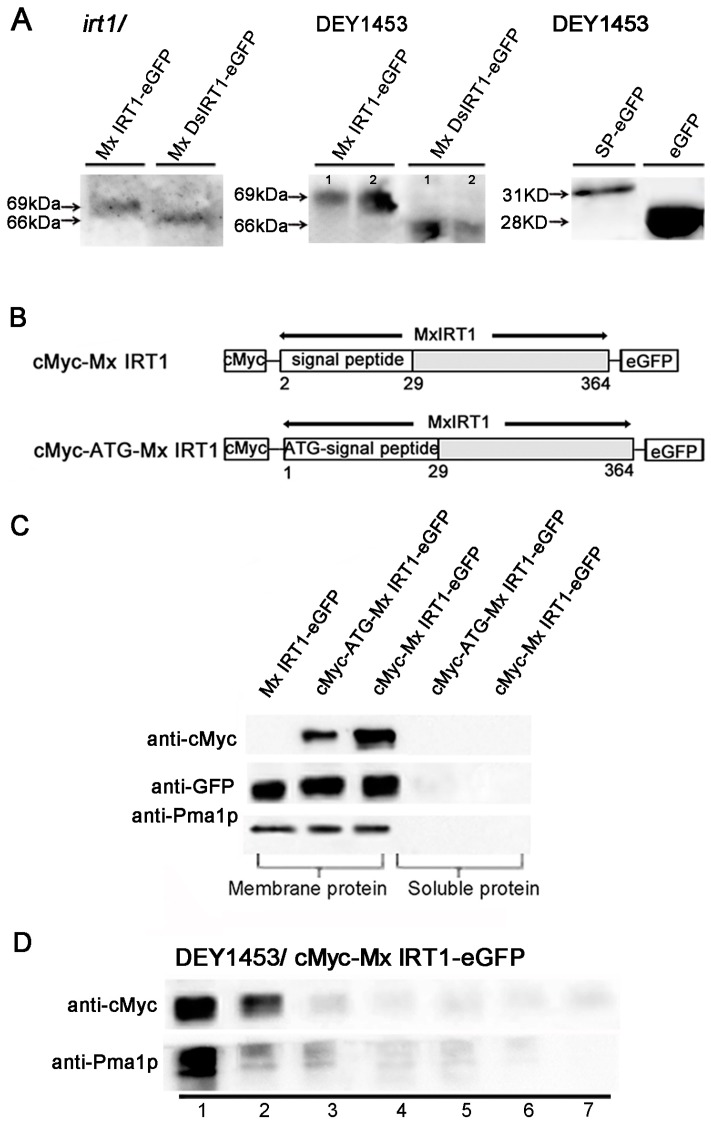
Detection of the SP using the cMyc-antibody in different fractions. (**A**) SDS-PAGE immunoblot analysis of the Mx IRT1-eGFP, Mx DsIRT1-eGFP, SP-eGFP, and eGFP in the total protein from transgenic *Arabidopsis irt1* and yeast DEY1453 using a monoclonal GFP antibody; line **1** and **2** in DEY1453/Mx or Ds IRT1-eGFP represented the experimental repeat; (**B**) Schematic diagrams of the constructs of cMyc-Mx IRT1-eGFP and cMyc-ATG-MxIRT1-eGFP used for expression in yeast cells; (**C**) Immunoblot detection of cMyc *N*-terminal tagged Mx IRT1 constructs in the membrane fraction using a monoclonal cMyc antibody and an anti-Pma1p antibody to detect the membrane fraction; and (**D**) Analysis of cMyc-Mx IRT1-eGFP fusion proteins in the different fractions.

For most ER target proteins, the *N*-terminal SP is cleaved from the protein by the ER-resident signal peptidase while it is still growing on the ribosome. If the functional *N*-terminal SP is in fact cleaved from mature Mx IRT1, then the Mx IRT1-eGFP and Mx DsIRT1-eGFP proteins from the expression constructs should co-migrate when separated by SDS-PAGE (dodecyl sulfate, sodium salt Polyacrylamide gel electrophoresis). Unexpectedly, the mature MxIRT1-GFP showed a higher molecular weight than Mx DsIRT1-GFP when purified from the transgenic plants and yeast. By extending the time of SDS-PAGE electrophoresis for clear separation, it was found that the protein band for Mx IRT1-eGFP was approximately 3 kDa larger than that of Mx *Ds*IRT1-eGFP (69 *vs.* 66 kDa, respectively) in both plant and yeast cells (Figure 6A). Meanwhile, we compared the molecular weight of SP_(1–54)_-eGFP with ΔSP-eGFP_(30–54)_ in transformed yeast. Per the molecular weight, the SP_(1–54)_-eGFP (~31 kDa) was ~3 kDa larger than ΔSP-eGFP_(30–54)_ (28 kDa) (Figure 6A). These results provide evidence that the signal peptide of the Mx IRT1 is not cleaved from the mature protein.

For further evidence that the *N*-terminal SP is retained on the mature protein, a cMyc-tag was added to the *N*-terminus end of Mx IRT1, upstream of the SP region. Two constructs were prepared for expression in yeast (Figure 6B). The first, designated pYES-cMyc-Mx IRT1-eGFP, lacked the Mx IRT1’s ATG initiation codon. The second, designated pYES-cMyc-ATG-Mx IRT1-eGFP, contained the initiator ATG codon (Figure 6B). These were transformed into yeast cells for analysis of *N*-terminal cleavage. Proteins were extracted by phase partitioning into two distinct fractions (a reagent kit “Thermo Mem-PER Eukaryotic Membrane Protein Extraction”), a hydrophilic fraction containing soluble protein and a hydrophobic fraction that included the membrane proteins. The presence or absence of the *N*-terminal sequence was assayed by immunoblot analysis using a monoclonal anti-cMyc antibody, as well as a monoclonal anti-GFP antibody. As shown in Figure 6C (top row), the anti-Myc detected both of the the *N*-terminal cMyc-tagged proteins (cMyc-ATG-Mx IRT1-eGFP and cMyc-Mx IRT1-eGFP) in the membrane fraction, and not in the soluble fraction. As a positive control, anti-GFP detected all three proteins, including Mx IRT1-eGFP (without the cMyc-tag, as a positive control) only in the membrane fraction (Figure 6C, middle row). Similarly, the PM marker Pma1p was found only in the membrane fraction and not in the soluble component (Figure 6C, bottom row). We also extracted intact membrane fractions from yeast transformed with pYES-cMyc-Mx RT1-eGFP using an Optiprep density gradient described before and detected cMyc using the cMyc antibody. As shown in Figure 6D, cMyc signals were mainly present in fractions 1 and 2, similar to Pma1p. This result further demonstrates that the SP is present on the mature Mx IRT1. Taken together, the data shown in Figure 6 support that the *N*-terminal region of Mx IRT1, shown in this study to be a bona fide functional SP, remains intact on the mature PM-associated Mx IRT1 molecule.

Since the discovery of SPs, the SP hypothesis of secretory proteins has predicted the co-translational targeting, processing, and removal of the *N*-terminal signaling sequence, a process mediated through cytoplasmic chaperones such as HSP70/40 that promote SP interactions with a signal-recognition particle located within the rough ER membrane [26,27,28,29]. In addition, a better understanding of the mechanisms by which integral proteins interact with the ER was provided [30]. Integral membrane proteins located in the PM, Golgi, lysosomes (tonoplast), and peroxisomal membrane are all synthesized on the rough ER membrane. Numerous studies have demonstrated a paradigm in which *N*-terminal SPs of PM integral proteins were cleaved by signal peptidases during translocon-mediated insertion into the ER membrane [13,18,25]. However, there are some exceptions, such as sucrase-isomaltase (type II) [31], prion protein [32], CD18, and the β-subunit of β_2_-integrins [14], in which the SP has been found to be uncleaved. In this study, we presented another exception to the canonical pathway [30] by showing that an essential plant membrane protein, Mx IRT1 has a non-cleavable SP protein that mediates its entry into the ER and trafficking to the PM, thereby enabling its critical iron transport function.

It is not yet known why some PM proteins require that an uncleaved SP be retained at their *N*-terminus, whereas most others do not. Recent work suggests that the uncleaved SPs do not only serve the classical mediators of ER targeting, but might in fact have additional role(s) as well. As one example, the uncleaved SP of the PM protein CD18 has been found to be a factor in rendering ruminants susceptible to leukotoxin of the pathogen *Mannheimia haemolytica* [14]. As to the uncleaved SP of Mx IRT1, we can speculate that it might play additional roles as well, such as participating in regulation of the protein stability, maintaining correct the protein location, mediating interaction with other proteins, or in binding of as yet unknown ligands. Our results also suggest that Mx IRT1 likely possesses nine TMDs instead of eight. Interestingly, our results also suggest that Mx IRT1 likely possesses nine TMDs instead of the predicted eight, possibility indicating an insertional or positional role for the attached SP peptide.

To date, the identification of the *N*-terminus of IRT1 family members as a functional SP has only been predicted based on sequence analysis. In this study, we have experimentally analyzed properties of the Mx IRT1 of the *N*-terminus and shown it to be a bona fide signal peptide for ER sorting, a property shared with other well characterized ER markers such as the signal peptide of *Arabidopsis* wall-associated kinase 2 [22]. What makes Mx IRT1 unique from other known plant PM proteins is its uncleaved *N*-terminal SP peptide. Consistent with our localization experiments, the lost of its correct transport capability for the truncated Mx IRT1 protein was due to its improper trafficking and localization within the cell. We found that the full-length Mx IRT1 could rescue the growth of *Arabidopsis IRT1* mutant, but Mx DsIRT1, which lacked the SP, could not. Interestingly, immunoblot analysis detected both the full-length Mx IRT1 as well as the truncated version of Mx DsIRT1 in transgenic *Arabidopsis IRT1*, indicating that there are several other factors that contribute to the lack of degradation of the truncated Mx DsIRT1. With the yeast heterologous complementation experiments, we verified the ability of Mx IRT1 to transport bivalent iron and cadmium, as has been demonstrated for the related iron transport protein At IRT1 [2,33,34]. Recently, there has been some controversy related to the functional necessity of the His-box region in the At IRT1 and Mx IRT1 proteins. The His-box has been shown to be essential for iron transport in Mx IRT1, but not in At IRT1 [35,36]. Therefore, the exact mechanism of divalent metal transport utilized by the IRT family members warrants further study. Based on findings presented here, it would be highly interesting to determine if the attached SP sequence of MxIRT1 plays a role in mediating the metal absorption efficacy of this important PM-localized iron transport protein.

## 3. Experimental Section

### 3.1. Construction of Expression Plasmids for Yeast and Plants

Plasmid pGEM-T-Mx *IRT1*, containing the entire coding sequence of Mx *IRT1*, was used as the original template for preparation of all other constructs. The SP-deleted Mx *DsIRT1* was amplified from this template using an Mx *DsIRT1* forward primer (Mx *Ds*IRT1-F, downstream of the SP coding region) and an Mx *DsIRT1* reverse primer (Mx *Ds*IRT1-R; see Table S1 for primer sequences). Both the intact and deleted sequences were then cloned into the pMD18-T vector (TaKaRa Bio, Tokyo, Japan).

For expression in yeast cells, Mx *IRT1* and Mx *DsIRT1* were subcloned from pMD18-T into pDBLeu using the SalI and SpeI. For the GFP fusion protein Mx IRT1-eGFP, the eGFP sequence from pEGFP (Clontech, Palo Alto, CA, USA) was fused with Mx IRT1 using BamHI and NotI to form pYES-Mx *IRT1*-eGFP (XbaI-Mx *IRT1*-BamHI-*eGFP*-NotI). For the Mx *DsIRT1*-eGFP, the *DsIRT1* fragment was amplified using the primers XbaI-Mx *DsIRT1*-F and BamHI-IRT-R. Using the XbaI and BamHI, Mx *DsIRT1* was subcloned into pYES-eGFP. For the SP-eGFP fusion, sequence encoding the signal peptide of Mx *IRT1* cDNA was amplified and subcloned into pYES-eGFP using the primers HindIII-SP-F and BamHI-SP-R.

For transient and stable expression in plant cells, Mx *IRT1*-eGFP fragment was cloned into pGEM-T using XbaI and NotI, which added SphI and SacI sites to the insert. The resulting SphI-XbaI-Mx *IRT1*-BamHI-EGFP-NotI-SacI fragment was then cloned into pBI221 (Clontech). A HindIII-35S_pro_-XbaI-Mx IRT1-BamHI-eGFP-SacI-NOS_terminator_-EcoRI fragment was cloned into pCAMBIA1301 for stable plant transformation. pBI221-Mx *DsIRT1*-eGFP was constructed using XbaI-DsMxIRT1-BamHI (from pMD18T-DsMxIRT1) to replace the Mx IRT1 fragment that was present in this vector. pCAMBIA-Mx *DsIRT1*-eGFP was constructed using the same strategy as pCAMBIA-Mx *IRT1*-eGFP. For pBI221-SP-eGFP construction, the SP fragment of Mx *IRT1* cDNA was amplified the primers XbaI-SP-F and BamHI-SP-R, and then subcloned from pBI221-Mx *IRT1*-eGFP into pBI221-eGFP. Construction of pBI221-ΔSP-eGFP used the forward primer XbaI-ΔSP-F and the reverse primer BamHI-SP-R. To make pBI221-SP-TM1-eGFP construction, the forward primer was XbaI-SP-F, and the reverse primer was BamHI-SP-TM1-R.

### 3.2. Yeast Strains and Functional Expression

Yeast mutants DEY1453 (*MATa/MATα ade2/+ can1/can1 his3/his3 leu2/leu2 trp1/trp1 ura3/ura3 fet3-2::HIS3/fet3-2::HIS3 fet4-1::LUE2/fet4-1::LUE2*) (the *fet3fet4* double mutant) and DY1457 (MATα ade6 can1 his3 leu2 trp1 ura3) were generous gifts from David Eide (Department of Nutritional Sciences, University of Wisconsin, Madison, WI, USA). For the metal accumulation assay, functional expression and localization analysis, the plasmid constructs were transformed into appropriate yeast mutant strains. Transformants were grown and selected on an SD medium lacking uracil (SD-Ura). Transformation and functional tests were performed as previously described [7].

### 3.3. Metal Accumulation Assay

For the iron content assays, the yeast cells were cultured in YPD (yeast extract peptone dextrose) media liquid medium with shaking for 1–2 days. After pre-culture to OD_600_ = 1, the cells were transferred to uptake medium (YPD medium with added concentrations of metal ions as specified in text and figure legends) and cultured for 4 h at 28 °C with shaking. The cells were then collected by centrifugation at 9000 rpm for 5 min and washed 3 times with SC. After removal of the final supernatant liquid, 1 mL of deionized water was added to the pellet along with a volume of snail enzyme (snailase) sufficient to give a final concentration of 5 μg/mL. This product was then incubated for 60 to 90 min with continuous shaking. To prepare the yeast cell extracts, the cell preparations were boiled for 10 min and then centrifuged at 9000 rpm for 10 min. Deionized water was added to bring the volume of the resulting pellet to 8.0 mL. The iron and cadmium concentration in the resulting yeast cells was measured by Inductively Coupled Plasma Mass Spectrometry (ICP-MS) using a PerkinElmer Optima 2000 DV (PerkinElmer Inc., Wellesley, MA, USA).

### 3.4. Plant Growth Conditions

The plant transformation was performed as described previously [35]. Wild type seeds of *Arabidopsis* (ecotype Columbia; cat. No. WT-02-17, Lehle seeds, Round Rock, Tucson, AZ, USA *gl-1*), *irt1-1* [35] and T_2_ transgenic seeds were sterilized, placed in the dark at 4 °C for 2 days, and then sown on 1/2 MS medium plates supplemented with 2% sucrose, 1 mM MES, and 1% agar, pH 5.8. Transgenic plants were grown on plates supplemented with hygromycin (20 g/mL). Plates were incubated at 23 °C for 7 days. Seedlings were transferred to the normal 1/2 MS medium plates or the iron deficient 1/2 MS medium plates for 10 days. To observe the germination of different T_3_ transgenic lines, the seeds were placed in the dark at 4 °C for 2 days and then sown on the iron deficient 1/2 MS medium for 5 days.

### 3.5. Fluorescent Imaging of Subcellular Localization in Rice Protoplasts and Yeast Cells Using Laser Scanning Confocal Microscopy

Fluorescence constructs that were expressed in either yeast cells or rice leaf/suspension protoplasts are described here or described previously [7,37,38]. Fluorescent imaging and analysis was performed using a Carl ZEISS 5 Live Laser Scanning Confocal Microscopy system (Zeiss, Oberkochen, Germany) and ZEN software (Zeiss). After an overnight incubation in the dark, GFP (green fluorescence proten), mCherry-PM (red fluorescence), FM4-64, mCherry-ER (red fluorescence), and ER-Tracker™ Dyes Blue-White DPX (Invitrogen, Carlsbad, CA, USA) signals were examined under a confocal microscope at excitation wavelengths of 488, 561, 561, 561 and 405 nm, respectively. The mCherry-ER vector, named ER-rk, was created by combining the signal peptide of *Arabidopsis* wall-associated kinase 2 (At WAK2; stock number: *CD3-9*59) [22]. And the mCherry-PM was based on the full-length coding region of At PIP2A (stock number: *CD3-1007*) [22].

### 3.6. Isolation of Proteins from Yeast and Arabidopsis

The reagent kit The Thermo Mem-PER Eukaryotic membrane Protein Extraction (Prod#89826, Thermo Fisher Scientific Inc., Rockford, IL, USA) was used to purify the membrane fraction from yeast cells. Yeast cells cultivated under the conditions described above were lysed with 2 mg/mL snailase in sorbitol-buffer (1 M d-Sorbitol, 0.1 M EDTA-Na_2_, pH 7.4, 0.1% (*v*/*v*) β-Mercaptoethanol) at 37 °C for 1 h and washed with PBS [39]. To improve the solubilization of membrane proteins in the total protein extracts, the yeast cells, lysed with snailase, were resuspended in RIPA buffer [8] (50 mM Tris−HCl, pH 7.5, 150 mM NaCl, 0.5% sodium deoxycholate, 1% Nonidet P-40, 0.1% SDS, and protease inhibitor cocktail mixture) for 2 h on ice. After centrifugation at 15,000× *g* for 45 min at 4 °C, the supernatant was collected and prepared for immunoblotting. Isolation of PM and *E*/*V* fractions from yeast cells was performed according to methods described in previous studies [40]. Cells were lysed and vortexed with glass beads in buffer L (25 mM Tris, pH 8, 2.5 mM EDTA). The lysates were centrifugated (700× *g* for 10 min) to clear the unbroken cells and subjected to differential centrifugation at 20,000× *g* for 20 min. The supernatants were discarded, and the pellets were resuspended in buffer B (10 mM Tris, pH 7.4, 0.2 mM EDTA, 0.2 mM DTT (dithiothreitol)), and 0.5 mL was loaded on top of a sucrose-step gradient (0.5 mL 53%, 1 mL 43%, in buffer B). After centrifugation (2 h at 100,000× *g* in TLS55 rotor), six fractions were collected from the top.

For the isolation of intact membrane fraction from yeast cells, the Optiprep density gradient centrifugation method was used according to methods described in previous studies [23,24], however, the detergent triton X-100 was not added in this study (Figure 6C). Yeast were washed in water, collected, and then dissolved in TNE buffer (50 mM Tris−HCl, pH 7.4, 150 mM NaCl, 5 mM EDTA) containing a protease inhibitor cocktail mixture by vortexing with glass beads at 4 °C. By centrifugation at 500× *g* for 5 min, the supernatant was collected on ice. The mixed lysate was adjusted to a final concentration of 40% Optiprep with a solution of 60% Optiprep (D1556-250ML, Sigma–Aldrich, St. Louis, MO, USA) and then overlaid with 30% Optiprep in TNE and TNE. The samples were centrifuged at 46,000 rpm for 4 h in a TLS55 rotor (Beckman Coulter, Miami, FL, USA), and 7 equal fractions were collected. Every fraction was prepared for SDS-PAGE and immunoblot.

Total proteins were prepared from the roots and shoots of wild type and transgenic *Arabidopsis* according to methods described in previous studies [35].

### 3.7. Immunoblotting Analysis

Proteins, including membrane, soluble and total proteins, were separated by SDS-PAGE [41] and transferred to a PVDF (polyvinylidene difluoride) membrane by electroblotting [42]. Immunodetection was performed using the following antisera: a GFP mouse monoclonal antibody diluted 1:1000 (Santa Cruz Biotechnology: sc-9996, Santa Cruz, CA, USA), a cMyc-tag rabbit monoclonal antibody diluted 1:1000 (Cell Signaling Technology: 71D10, Danvers, MA, USA), a Pma1p mouse monoclonal antibody diluted 1:1000 (Santa Cruz Biotechnology: sc-57978), and rabbit polyclonal anti-peptide At IRT1 antibody diluted 1:5000. After incubation with the appropriate antibody, the membranes were washed with TBST. Depending on the primary antibody used, the blots were then incubated with either goat anti-rabbit polyclonal secondary antibody conjugated to horseradish peroxidase (HRP) (ab97051, Abcam Inc., Cambridge, MA, USA) diluted 1:5000, or else anti-mouse IgG conjugated to HRP (Santa Cruz Biotechnology, sc-2005) diluted 1:5000. The signal of specific proteins was detected with chemiluminescent substrate (ECL™ Prime, GE Healthcare, RPN2232, Chalfont St Giles, UK).

## 4. Conclusions

The findings presented here clearly demonstrate that Mx IRT1, an iron transport protein essential for plant growth, viability, and productivity, is a multipass PM protein that must be sorted to the PM via an SP-dependent ER trafficking process. When the *N*-terminal signal sequence was deleted, the SP deficient Mx DsIRT1 protein could not be translocated into the ER for trafficking to the PM. Unlike the intact Mx IRT1 protein, which showed strong specific localization to the PM in both plant and yeast cells, the truncated protein showed a diffuse pattern of accumulation throughout the cytosol of both cell types, indicating mis-localization due to the lack of a functional SP. Indicative of improper trafficking, Mx DsIRT1 expressed in either *Arabidopsis irt1* or yeast mutants deficient in iron uptake could not rescue metal uptake activity, whereas the intact Mx IRT1 protein could restore efficient iron uptake capability to these cells. Surprisingly, immumoblot analysis demonstrated that the *N*-terminal SP was not cleaved from the mature Mx IRT1 protein in either cell type. Taken together, these results support the concept that the *N*-terminal SP is a critical component necessary for moving the newly synthesized Mx IRT1 into the correct PM-targeting ER sorting pathway. This study defines the *N*-terminal region of Mx IRT1 as a novel uncleaved SP that is essential for Mx IRT1 production, trafficking, and iron uptake capability in plants.

## Accession Number

Sequence data for Mx IRT1 can be found in the GenBank data library under accession number AY193886.

## Supplementary Materials

Supplementary tables can be found at http://www.mdpi.com/1422-0067/15/11/20413/s1.
